# Organizing Alzheimer’s Disease Endophenotypes Into Subdivisions of Biological Domains Improves Therapeutic Target Hypothesis Selection

**DOI:** 10.1002/alz.092649

**Published:** 2025-01-03

**Authors:** Stephen Keegan, Gregory A. Cary, Jesse C Wiley, Karina Leal, Robert R. Butler, Anna Greenwood, Gregory W Carter, The Emory‐Sage‐SGC TREAT‐AD Center

**Affiliations:** ^1^ The Jackson Laboratory, Bar Harbor, ME USA; ^2^ Sage Bionetworks, Seattle, WA USA; ^3^ Stanford University, Stanford, CA USA

## Abstract

**Background:**

Alzheimer’s disease (AD) therapeutics have largely been unsuccessful in alleviating disease burden in those afflicted by the disease. The TREAT‐AD Consortium is an international group of academic researchers dedicated to identifying novel molecular targets for AD from underexplored areas of disease linked pathology.

**Method:**

Utilizing a top‐down expert curation approach of organizing Gene Ontology terms into endophenotypes of AD, we developed 19 biological domains. To provide more functional insight of these biological domains impacts on AD, we recreated the process to make 80 subdomains. The biological domain describes the endophenotype, the subdomain describes the important functions of that endophenotype. The gene lists of these clusters are turned into protein‐protein interaction networks.

**Result:**

Using the structures of the Gene Ontology, biological domains, and subdomains, it’s possible to identify a genes relationship to an endophenotype and its impact on other genes implicated in AD. Analyzing your target at a biological domain level connects endophenotypes and at the subdomain level it connects the functions that lead to the endophenotype.

**Conclusion:**

A layered knowledge approach of investigating the impact a gene has on an endophenotype provides insight into how a potential therapeutic target would interact with other areas of disease biology. TREAT‐AD investigators will validate these targets in cell models and generate experimental resources to support further target development.

